# Negative Differential Resistance Effect in Ru-Based RRAM Device Fabricated by Atomic Layer Deposition

**DOI:** 10.1186/s11671-019-2885-2

**Published:** 2019-03-11

**Authors:** Yulin Feng, Peng Huang, Zheng Zhou, Xiangxiang Ding, Lifeng Liu, Xiaoyan Liu, Jinfeng Kang

**Affiliations:** Institute of Microelectronics, Peking University, Beijing, 100871 China

**Keywords:** Negative differential resistance, Ruthenium, RRAM, Atomic layer deposition

## Abstract

In this work, Ru-based RRAM devices with atomic layer deposited AlO_y_/HfO_x_ functional layer were fabricated and studied. A negative differential resistance (NDR) behavior was observed during the voltage set process, and its physical origin was explored. Based on the physics understanding of the resistive switching, the measured NDR behavior is believed to be associated with the partially unipolar reset effect, which is due to the recombination between oxygen vacancies and the thermally released oxygen ions from the RuO_2_ interface layer. The measured electrical characteristics and X-ray photoelectron spectroscopy (XPS) results verified the physical interpretation.

## Introduction

As one of the most promising emerging non-volatile memories, resistive random-access memory (RRAM) has been extensively studied regarding material optimization, performance improvement, and device integration [1–4]. Due to the significant advantages such as simple cell structure, fast operational speed, low power consumption, and incomparable miniaturization potential [5], RRAM has been widely applied in brain-inspired neuromorphic computing and reconfigurable Boolean logic [6–10] and also exhibits great potential for storage class memory (SCM) applications [11]. However, as indicated by the International Roadmap for Devices and Systems 2017 (https://irds.ieee.org/images/files/pdf/2017/2017IRDS_ES.pdf), challenges including scalability, device reliability, and process compatibility are still hindering RRAM’s developments. Therefore, construction of CMOS-compatible RRAM devices with superior performance is of great significance to applications that are based on the 1T1R structure of RRAM cell [12–14]. According to a previous study [15], a Ru-based RRAM device shows great potential compared with Pt-based ones, whereas negative differential resistance (NDR) was demonstrated during a voltage set process. The NDR phenomena accompanied with resistive switching have been investigated in other RRAM structures, which were mainly due to the trap/detrap of electronic carriers between deeply localized states induced by implanted metal nanoparticles [16, 17], or the accumulation of defects caused junction reinstallment [18]. While the appearance of NDR in Ru-based RRAM cell under large current is still pendent, in this work, the electrical performance of a Ru-based RRAM device fabricated by atomic layer deposition (ALD) technique was evaluated. Based on the X-ray photoelectron spectroscopy (XPS) characterization and electrical measurements with different stimulus, the NDR phenomenon in the Ru-based RRAM can be explained in the framework of the oxygen vacancy conductive filament model.

## Methods

**Fig. 1:**
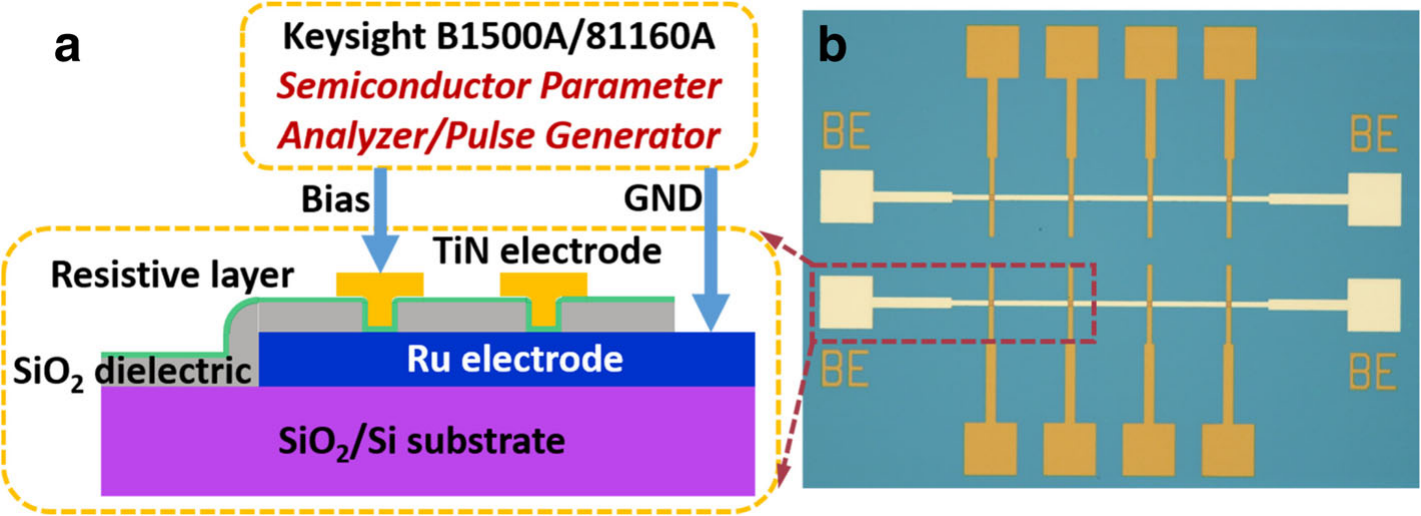
**a** Schematic diagram of a Ru-based device. **b** Fabricated RRAM array

The schematic diagram of the device structure and fabricated RRAM array are shown in Fig. 1a, b, respectively. The Ru/AlO_y_/HfO_x_/TiN RRAM device was fabricated on a thermally oxidized 300-nm SiO_2_ substrate. An 80-nm Ru thin film was deposited on a 20-nm Ti adhesion layer as a bottom electrode by DC magnetron sputtering (Angstrom Engineering NEXDEP) using a Ru metal target. SiO_2_ grown by plasma-enhanced chemical vapor deposition (PECVD) serves as dielectric to isolate electrodes and forms the vias. Then the resistive layer of 2 nm AlO_y_ and 3 nm HfO_x_ was deposited by an atomic layer deposition system (PICOSUN R200) at 300 °C with trimethylaluminum (TMA) and tetrakis[ethylmethylamino]hafnium (TEMAH) precursors. After that, 80 nm TiN was reactive sputtered using a Ti target and etched to form the top electrode patterns. Another dry etching was also performed to expose the bottom electrode for electrical contact.

The element analysis was performed by a X-ray photoelectron spectroscopy (XPS) system (Thermo Scientific ESCALAB 250Xi) using the fresh sample without a top electrode. Prior to the measurement, surface cleaning was conducted with Ar plasma before measurement to eliminate the influence of C. The peak position of Al 2p was used for calibration. The electrical measurements were performed at room temperature by a semiconductor device analyzer (Agilent B1500A) and pulse function arbitrary generator (Agilent 81160A).

## Results and Discussion

**Fig. 2:**
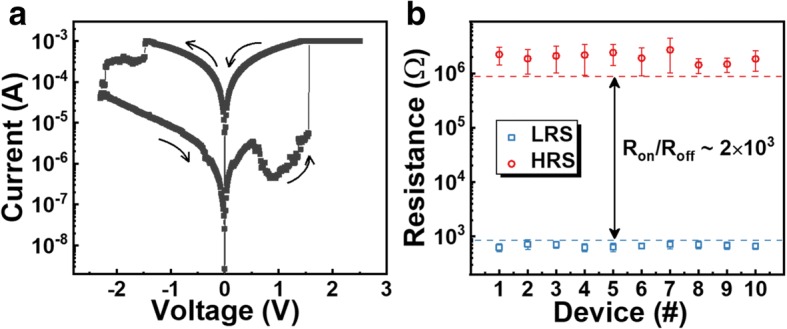
**a** DC characteristics. **b** HRS/LRS statistical distributions of 10 Ru-based RRAM devices

The typical IV characteristics of Ru-based RRAM devices are shown in Fig. 2a. After electroforming, a positive voltage (2.5 V) was applied for set process to switch the cell from high-resistance state (HRS) to low-resistance state (LRS) with a compliance current of 1 mA to prevent the permanent breakdown during the conductive filament (CF) formation. After the set transition, a negative voltage (− 2.3 V) was used to switch the device from LRS to HRS with a gradual decreasing current. In order to evaluate the resistance variability from device to device, 10 Ru-based RRAM cells were chosen arbitrarily. As depicted in Fig. 2b, the statistical results demonstrate the excellent uniformity of HRS and LRS with a resistance window larger than 10^3^, which could be a promising candidate for NVM-based logic applications. Compared to the previously reported Pt-based devices [2], it is worth noting that a NDR phenomenon was observed during the set process, where the current decreases with the increasing voltage (set-phase 1) in a limited voltage scale followed by increasing to compliance current (set-phase 2).

**Fig. 3:**
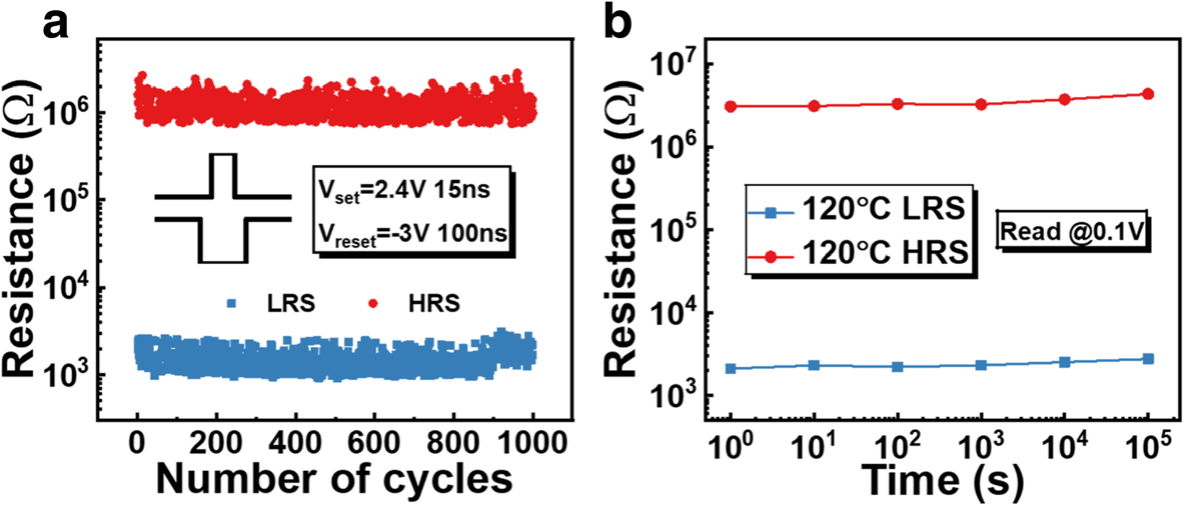
**a** 1000 endurance cycles. **b** High temperature retention behavior of a Ru/AlO_y_/HfO_x_/TiN RRAM device

The cycle-to-cycle variability of Ru-based RRAM devices was also investigated under pulse mode to study the cycling uniformity. The pulses for set (2.4 V, 15 ns) and pulse (− 3 V, 100 ns) are used to switch the device between LRS and HRS with a read voltage of 0.1 V after each pulse. As shown in Fig. 3a, the device of 1000 cycles has a uniform distribution with standard deviations of 379 Ω and 3 × 10^5^ Ω for LRS and HRS, which results in a stable memory window larger than 100. No endurance degradation occurs even after 10^6^ switching cycles as previously reported in Ref. [15]. In addition, the device also demonstrates an excellent retention property as shown in Fig. 3b. Both LRS and HRS resistance maintain a constant value over 10^5^ s at 120 °C without failure.

**Fig. 4:**
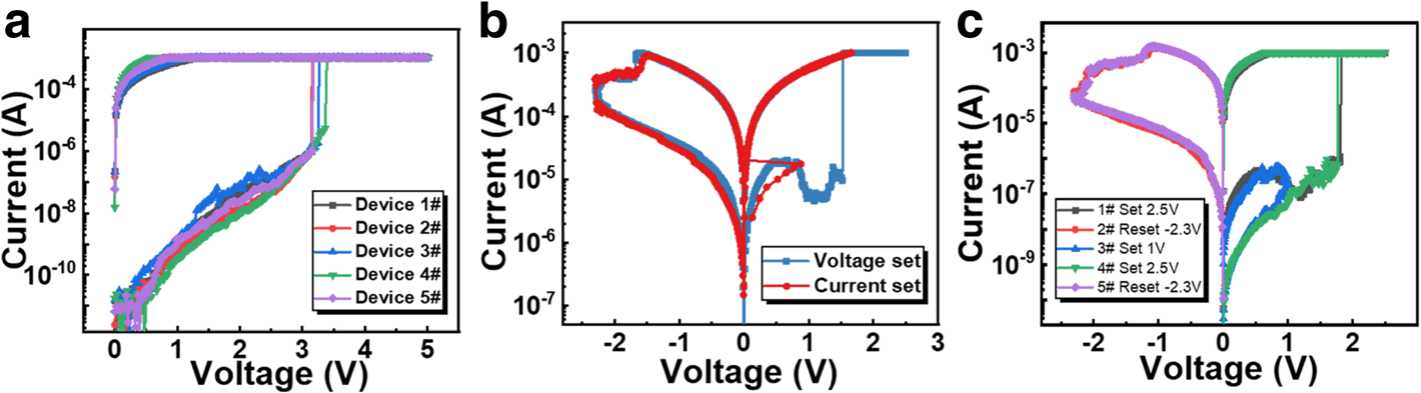
**a** Voltage forming of five Ru-based RRAM devices. **b** Voltage-driven and current-driven set processes in the same RRAM cell. **c** Incomplete set process with the stop voltage at the bottom of the current valley

In order to explore the NDR phenomenon, the *I–V* curves were measured in both voltage sweep mode and current sweep mode. Figure 4a displays the forming process of five randomly selected fresh RRAM cells. The current gradually increases followed by an abruption, indicating the formation of CF, while no NDR was observed. After electroforming, set operations in different sweep modes are conducted in the same cell in order to observe the current variation, as shown in Fig. 4b. For the current-driven set, the current increases slowly with a sudden decrease in voltage, demonstrating the transition of resistance from HRS to LRS. This behavior is distinct from the characteristic that is driven by voltage, which might be due to the different stimuli-induced Joule heating across the CF. Moreover, consecutive set/reset operations under different bias were performed to investigate the intermediate resistance state in the NDR region. An appropriate stop voltage of 1 V was applied during set process, as shown in Fig. 4c, to finish the sweep at the bottom of the valley. A non-volatile resistance state was obtained after the voltage was removed, which exhibited a unipolar resistive switching behavior. Therefore, this NDR phenomenon is tentatively attributed to a second reset of the CF during the set process.

**Fig. 5:**
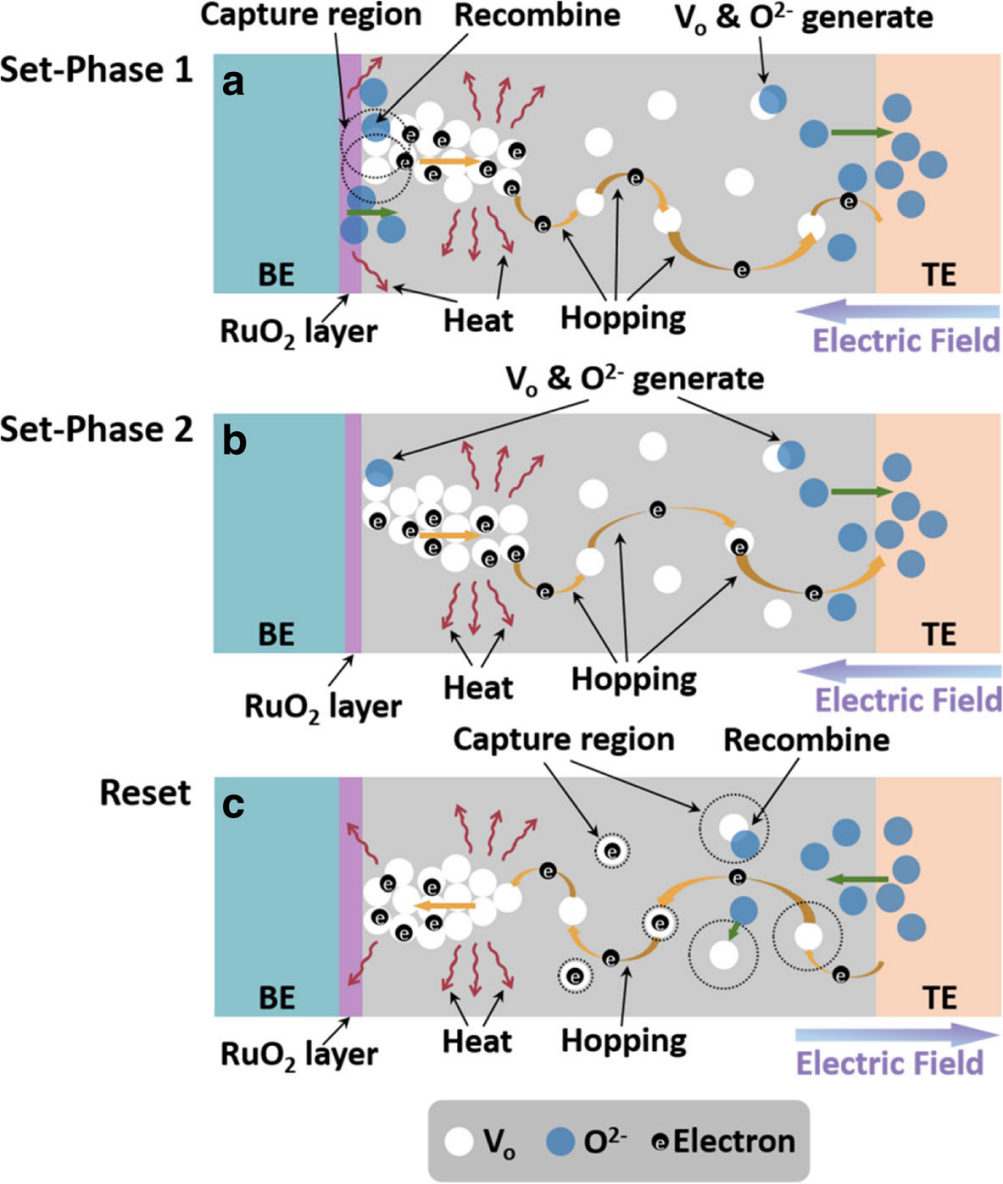
Physical processes of resistive switching in Ru-based RRAM. **a** NDR effect (set-phase 1). **b** Common SET (set-phase 2) processes. **c** RESET process of the device

Combined with the measurements in different modes and device fabrication processes as well as the properties of RuO_2_, the physical origin of the NDR phenomenon was proposed, as illustrated in Fig. 5. A previous study [19, 20] suggested that the generation and recombination of electron depleted oxygen vacancy (*V*_o_) and oxygen ion (O^2−^) under different electric polarities are responsible for the conventional HfO_x_-based bipolar RRAM devices, which is similar to the set-phase 2 and reset processes. However, unlike the conventional set process, a second rupture of the CF takes place disconnecting the Ru electrode and CF and leading to the NDR. In general, the oxygen atoms dissociate into *V*_o_ and O^2−^ under electric field with the drifting of the O^2−^ to the top electrode, leaving the *V*_o_ to form the CF that is used for electron transport. But due to the Joule heating caused by the electric field, the formed RuO_2_ interface layer would slowly decompose at ~ 600 °C and releases O^2−^ which could recombine with the electron depleted *V*_o_ (*V*_o_^2+^) near the Ru electrode (set-phase 1) [21], resulting in a current decrease. This process can also be viewed as a partial unipolar reset process. With the further increasing voltage, the CF between TE and BE will be reconstructed by an accumulation of *V*_o_ as shown in set-phase 2 and RRAM cell switches to LRS. During reset, two processes take place simultaneously: (1) the O^2−^ released from TiN electrode rapidly recombine with the positively charged *V*_o_ because of the enhanced capture section, (2) the O^2−^ drifting toward BE reacts with Ru and reforms the RuO_2_ interface layer due to the local Joule heating [22]. At this condition, the RRAM cell switches to HRS.

**Fig. 6:**
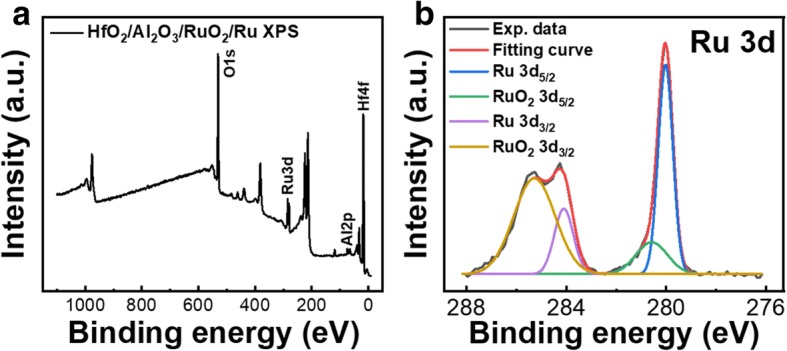
XPS spectra of **a** Ru/AlO_y_/HfO_x_ thin film and **b** Ru 3d core level. The RuO_2_ thin film between Ru and AlO_y_ forms during ALD process [18]

XPS analysis of the RuO_2_ interface layer, which was formed during the device fabrication process, also supports the proposed explanation of the NDR effect. Figure 6a shows the XPS full spectrum of the sample, which includes O 1s, Ru 3d, Al 2p, and Hf 4f core levels. The other unmarked peaks all correspond to these elements with different electron orbits. The fitting curve in Fig. 6b fits perfectly with the experimental data and is divided into four peaks, which correspond to the Ru 3d_5/2_ (280.01 eV for Ru and 280.75 eV for RuO_2_) and Ru 3d_3/2_ (284.3 eV for Ru and 285.26 eV for RuO_2_) core levels, demonstrating the coexistence of the Ru and RuO_2_ in the thin film [23]. The low intensity of Ru 3d_5/2_ peak indicates that the formed RuO_2_ interface layer is very thin as we have expected.

## Conclusion

In this study, the CMOS-compatible Ru/AlO_y_/HfO_x_/TiN RRAM device was fabricated. The excellent switching performance was achieved with uniform device-to-device resistance and a large resistance window as well as a good high-temperature retention property. Based on the electrical measurements and XPS analysis, the NDR phenomenon in the set process can be explained by the recombination of oxygen vacancies and oxygen ions released from the RuO_2_ interface layer due to the electric-induced Joule heating.

## Abbreviations

ALD: Atomic layer deposition
CF: Conductive filament
HRS: High-resistance state
LRS: Low-resistance state
NDR: Negative differential resistance
PECVD: Plasma enhanced chemical vapor deposition
RRAM: Resistive random-access memory
SCM: Storage class memory
XPS: X-ray photoelectron spectroscopy

## References

[1] Chen HY, Brivio S, Chang CC, Frascaroli J, Hou TH, Hudec B, Liu M, Lv HB, Molas G, Sohn J, Spiga S, Teja VM, Vianello E, Wong HSP (2017). Resistive random access memory (RRAM) technology: from material, device, selector, 3D integration to bottom-up fabrication. J Electroceram.

[2] Gao B, Chen B, Liu R, Zhang FF, Huang P, Liu LF, Liu XY, Kang JF, Chen HY, Yu SM, Wong HSP (2014). 3-D cross-point array operation on AlO_y_/HfO_x_-based vertical resistive switching memory. IEEE Trans Electron Devices.

[3] Yu SM, Chen HY, Gao B, Kang JF, Wong HSP (2013). HfO_x_-based vertical resistive switching random access memory suitable for bit-cost-effective three-dimensional cross-point architecture. ACS Nano.

[4] Wang XH, Wu HQ, Gao B, Li XY, Deng N, Qian H (2018). Thermal stability of HfOx-based resistive memory array: a temperature coefficient study. IEEE Electron Device Lett.

[5] Pan F, Gao S, Chen C, Song C, Zeng F (2014). Recent progress in resistive random access memories: materials, switching mechanisms, and performance. Mat Sci Eng R.

[6] Yu SM (2018). Neuro-inspired computing with emerging nonvolatile memory. Proc IEEE.

[7] Kang JF, Huang P, Gao B, Li HT, Chen Z, Zhao YD, Liu C, Liu LF, Liu XY (2016). Design and application of oxide-based resistive switching devices for novel computing architectures. IEEE J Electron Devices Soc.

[8] Han RZ, Huang P, Zhao YD, Chen Z, Liu LF, Liu XY, Kang JF (2017). Demonstration of logic operations in high-performance RRAM crossbar array fabricated by atomic layer deposition technique. Nanoscale Res Lett.

[9] Huang P, Kang JF, Zhao YD, Chen SJ, Han RZ, Zhou Z, Chen Z, Ma WJ, Li M, Liu LF, Liu XY (2016). Reconfigurable nonvolatile logic operations in resistance switching crossbar array for large-scale circuits. Adv Mater.

[10] Wu HQ, Yao P, Gao B, Wu W, Zhang QT, Zhang WQ, Deng N, Wu D, Wong HSP, Yu SM, Qian H (2017). Device and circuit optimization of RRAM for neuromorphic computing.

[11] Luo Q, Xu XX, Gong TC, Lv HB, Dong DN, Ma HL, Yuan P, Gao JF, Liu J, Yu ZA, Li JF, Long SB, Liu Q, Liu M (2017). 8-layers 3D vertical RRAM with excellent scalability towards storage class memory applications.

[12] Su YT, Chang TC, Tsai TM, Chang KC, Chu TJ, Chen HL, Chen MC, Yang CC, Huang HC, Lo I, Zheng JC, Sze SM (2017). Suppression of endurance degradation by applying constant voltage stress in one-transistor and one-resistor resistive random access memory. Jpn J Appl Phys.

[13] Liu HT, Lv HB, Yang BH, Xu XX, Liu RY, Liu Q, Long SB, Liu M (2014). Uniformity improvement in 1T1R RRAM with gate voltage ramp programming. IEEE Electron Device Lett.

[14] Wu W, Wu HQ, Gao B, Deng N, Yu SM, Qian H (2017). Improving analog switching in HfOx-based resistive memory with a thermal enhanced layer. IEEE Electron Device Lett.

[15] Feng YL, Huang P, Zhou Z, Zhu DB, Han RZ, Ding XX, Liu LF, Liu XY, Kang JF (2018). Ru-based oxide resistive random access memory for BEOL-compatible novel NVM applications.

[16] Wu FC, Si SY, Shi T, Zhao XL, Liu Q, Liao L, Lv HB, Long SB, Liu M (2018). Negative differential resistance effect induced by metal ion implantation in SiO_2_ film for multilevel RRAM application. Nanotechnology.

[17] Sun HT, Liu Q, Long SB, Lv HB, Banerjee W, Liu M (2014). Multilevel unipolar resistive switching with negative differential resistance effect in Ag/SiO_2_/Pt device. J Appl Phys.

[18] Guo T, Sun B, Zhou Y, Zhao HB, Lei M, Zhao Y (2018). Overwhelming coexistence of negative differential resistance effect and RRAM. Phys Chem Chem Phys.

[19] Huang P, Liu XY, Chen B, Li HT, Wang YJ, Deng YX, Wei KL, Zeng L, Gao B, Du G, Zhang X, Kang JF (2013). A physics-based compact model of metal-oxide-based RRAM DC and AC operations. IEEE Trans Electron Devices.

[20] Gao B, Wu HQ, Wu W, Wang XH, Yao P, Xi Y, Zhang WQ, Deng N, Huang P, Liu XY, Kang JF, Chen HY, Yu SM, Qian H (2017). Modeling disorder effect of the oxygen vacancy distribution in filamentary analog RRAM for neuromorphic computing.

[21] Campbell PF, Ortner MH, Anderson CJ (1961). Differential thermal analysis and thermogravimetric analysis of fission product oxides and nitrates to 1500°C. Anal Chem.

[22] Steeves MM (2011). Electronic transport properties of ruthenium and ruthenium dioxide thin films, The University of Maine.

[23] Song YH, Chen YL, Chi Y, Liu CS, Ching WL, Kai JJ, Chen RS, Huang YS, Carty AJ (2003). Deposition of conductive Ru and RuO_2_ thin films employing a pyrazolate complex [Ru(CO)_3_(3,5-(CF_3_)_2_-pz)]_2_ as the CVD source reagent. Chem Vap Depos.

